# Evaluation of Immunity to Measles in a Cohort of Medical Students in Rome, Italy

**DOI:** 10.3390/vaccines7040214

**Published:** 2019-12-13

**Authors:** Luca Coppeta, Giorgia Biondi, Piergiorgio Lieto, Antonio Pietroiusti

**Affiliations:** Department of Biomedicine and Prevention, University of Rome Tor Vergata, 00133 Rome, Italy; giorgia.biondi90@gmail.com (G.B.); piergiorgiolieto@gmail.com (P.L.); pietroiu@uniroma2.it (A.P.)

**Keywords:** measles, outbreak, vaccination, health care workers, students

## Abstract

Background: Measles is a highly contagious viral disease with serious complications. Currently, in Italy, measles vaccination is not mandatory for health care workers (HCWs) and medical students, and the free offer of the Measles Mumps Rubella (MMR) vaccine is the only national prevention measure to increase the coverage rate among these subjects. Aims: The aim of our study was to evaluate the impact on vaccination rate of the National Plan of Vaccine Prevention (NPVP) implemented in 2017. Material and Methods: This is a retrospective observational study that evaluated the measles-specific IgG immunity status of medical students at the University Tor Vergata of Rome, which underwent occupational health surveillance from 1 January to 31 December 2018. Results: In 2018, 84 of 319 students (26.30%) were serologically non-immune to measles; among these, 16 (19%) had previously been vaccinated, and 35 of the remaining 68 students accepted the MMR vaccine. Therefore, 33 out of 319 students did not undergo vaccination in 2018. These data are similar to those obtained in the previous year. In the 2017 screening, 84/314 (26.75%) students tested negative at the serological screening, whereas 15/85 (17.8%) among them documented a previous vaccination with two doses of the MMR vaccine; 69 students tested as unprotected. Vaccine compliance was 51.44%. Conclusions: No change in vaccination coverage occurred after the introduction of the last NPVP. Further efforts are needed to sensitize target populations about the relevance of vaccination; providing pre-employment screening for measles and free vaccine might be useful for this purpose.

## 1. Introduction

Measles is a highly contagious viral disease with serious complications: before vaccine introduction, more than 30 million cases and more than 2 million deaths occurred globally each year [1]. This disease is still one of the leading causes of death among young children worldwide, despite the availability of an effective and safe vaccine. The infection spreads from affected persons to susceptible hosts by airborne droplets, as well as by direct or indirect contact with nasal or throat secretions. Measles outbreaks can be extensive in communities where a large number of susceptible persons is present [2].

The World Health Organization (WHO) set the year 2020 as the target date for the eradication of measles in Europe, although according to epidemiological reports, measles infection continues to circulate in the European Region, due to suboptimal vaccination coverage (VC) and population immunity gaps: in 2018, over 83,000 measles cases and 74 deaths were reported [2,3]. In Italy, during the last year, 2526 cases and 8 deaths were referred [4]. 

The vaccine against measles has been available in Italy since 1979, and two doses are necessary for a protection of 99%. Despite the scientific evidence about vaccine safety and efficacy, the number of under-vaccinated or unvaccinated subjects has alarmingly increased in Italy starting from 2013 [5]. Then, a new National Plan of Vaccine Prevention (NPVP 2017–2019) was approved [6,7], followed, in the same year, by the Law 119/2017, which included measles vaccination among the mandatory vaccinations for children aged 0–16. It also specified some “at-risk” categories, among which health care workers (HCWs), in which case the vaccine may not only protect the HCWs but also avoid the spread of the disease to susceptible populations (i.e., patients). However, vaccination is strongly recommended but not mandatory for HCWs. The WHO estimates that about 59 million HCWs are potentially exposed every day to biological risks by working with infectious patients and coming into contact with contaminated fluids and materials [8].

The 2017–2019 Italian National Immunization Prevention Plan (INIPP) strongly recommends that HCWs be vaccinated [9], when presumptive evidence of immunity to measles (e.g., demonstrable immunization history) or serological evidence of protective antibodies are not available.

A study conducted in Italy in 2017 showed that 22.3% of all cases of measles infection occurred in hospital settings, and 6.6% of cases occurred among HCWs. [10]. A serological survey showed no measles-specific IgG antibodies in 4–10% of HCWs [11]. In another study, we found that the 12.90% of HCWs employed in a large University Hospital had a non-protective measles-specific IgG antibody titer [12].

Since the literature data reported that HCWs are at 2- to 19-fold more likely to develop measles infection than the general population [13,14], it is crucial to increase preventive activities, establish policies on the vaccination of HCWs, and involve hospital occupational health services in vaccine active supply [13].

Nevertheless, currently, measles vaccination is not mandatory for HCWs [7] and medical students in Italy, and the free offer of the Measles Mumps Rubella (MMR) vaccine along with an awareness campaign is the only prevention measure which has been implemented to increase the coverage rate among those subjects.

The aim of our study was to evaluate the immunity to measles among healthcare students at the University of Rome “Tor Vergata”, after the introduction of the new NPVP 2017–2019 and the free vaccine campaign.

## 2. Methods

This is a retrospective observational study, approved by the Ethical Committee for Research in Human Subjects of the PTV (Polyclinic Tor Vergata of Rome) Foundation (approval number 132/18). This study determined the immunity status to measles of medical students of the University who carried out health surveillance at the Occupational Medicine department from 1 January to 31 December 2018.

We measured measles-specific IgG antibodies in serum using a chemiluminescent test LIASON^®^ Measles IgG essay, (DiaSorin, Vercelli, Italy). The DiaSorin LIAISONa ^®^ Measles IgG assay uses a chemiluminescence immunoassay (CLIA) technology: sensitivity and specificity are 97% (95% CI: 91.7–99.4) and 93% (95% CI: 82.5–97.7), respectively. Data were extracted from the laboratory software database ModuLab^®^ (Werfen, Barcellona, Spain). Subjects showing a measles-specific IgG level higher than 16.5 AU/mL were considered serologically immune. Moreover, subjects who documented 2 doses of MMR vaccination were considered protected, despite having an antibody titer below 16.5 A /mL. For each subject, the following information was also collected: age, gender, date of vaccination, vaccination history.

Finally, we compared the results of our serological screening with data from the surveillance conducted in the year 2017 to assess if a change in vaccination coverage occurred.

Analyses were performed using the IBM SPSS software Version 23 (IBM Italia Spa, Milan, Italy). Results were considered statistically significant for a *p*-value threshold <0.05.

## 3. Results

This study included 319 subjects, 122 male and 197 female students in the medical area. The mean age was 25.7 years (SD 1.6). In our population, 84/319 (26.30%) students were serologically non-immune to measles. Comparing vaccination coverage for measles by sex, female students showed a higher vaccination rate than males (64.9% vs 35.1%, *p* = 0,024, Chi square test). Among students without vaccine coverage, 16 (19%) were previously vaccinated with two doses of measles vaccine and therefore were considered immune to the disease, even in the absence of a protective titer, in accordance with the Center for Disease Control (CDC) guideline [15]. The vaccination campaign was addressed to the remaining 68 subjects.

Among unprotected subjects, 35/68 (51.47%) accepted to be vaccinated with the MMR vaccine. Comparing vaccination compliance by sex, no significant difference was found. In order to evaluate the effectiveness of the national vaccination plan, we compared the results of our study with data from the serological screening performed during the previous year. In the 2017 screening, 84/314 (26.75%) students tested negative at the serological screening, whereas 15/85 (17.8%) among them documented a previous vaccination with two doses of the MMR vaccine; 69 students tested unprotected. During the 2017 occupational screening, vaccine compliance was 51.44%. We observed no difference in immune status, percentage of unprotected subjects, and vaccine compliance before and after the implementation of the national vaccination program (Table 1).

## 4. Discussion

The last National Plan of Vaccine Prevention (NPVP) 2017–2019 and the following Law 119/2017 approved in Italy in 2017 were aimed at increasing the vaccination coverage among the population. In children, VC rose from 87.26% in 2016 to 93.22% in 2018 [4]. An indirect parameter of the efficacy of vaccination may be represented by the incidence rate of measles, which in Italy dropped from 5.393 cases in 2017 to 2.526 in 2018 [16].

HCWs are at an increased risk of disease due to their occupational exposure; according to the vaccination plan, their adequate immunization is essential for the prevention and control of infections [7]. Medical students are in close and repeated contact with patients during their training, and the same vaccination recommendations targeting professionals apply also to them.

However, our study showed that the national vaccination campaign and the media informative actions performed in Italy after the 2017 infective outbreak did not increase immunity protection against measles among medical students: an almost identical percentage of unprotected subjects was observed in 2017 and 2018. Conflicting data on VC among medical students have been reported. In fact, a similar VC was found in a study on medical students performed in Lille, France: 78% of the medical students were covered for measles [17].

By contrast, a higher percentage of VC was reported in a study from Greece, with about 98,4% of medical and nursing students being immune to measles [18], and in a survey performed in Slovenia, showing 86.4% VC [19]. The reasons for these discordant results are unclear but might be related to differences in norms regarding vaccination among different countries.

The high percentages of unprotected medical students against measles underline the fact that in subjects born between 1992 and 1999, the immunity levels are inadequate, probably due to low MMR vaccination coverage (vaccination was not mandatory in those years) associated with a low chance of acquiring infection during childhood by unprotected children, because of the very low prevalence of measles infection in Italy during that period. The inadequate immunity coverage observed in subjects attending hospital settings increases the risk of nosocomial measles from affected patient to susceptible medical students.

In our study, the vaccination compliance was 51.47% in 2018 and 51.44% in 2017, and no difference was found by sex. By contrast, a survey conducted in 2017 reported male HCWs and medical students were more willing to be vaccinated with the MMR vaccine compared with female students [20]. Also, in this case no substantial difference was found before and after the NPVP implementation. The reasons why the attitude towards vaccination did not change in 2018 in comparison to 2017 (i.e., after the implementation of the NPVP) have not been explored in the current study. Future studies should investigate the causes of students’ hesitancy, in order to apply a focused campaign promoting vaccination in this group.

Workplace vaccinations represent a fundamental strategy to protect health and safety at work. Measles vaccination of medical students is important not only to reduce the burden of morbidity and mortality of patients but also to decrease the related remarkable costs for the National Health Service. The costs related to nosocomial infection involve direct costs, concerning the care of ill people, and indirect costs, mostly consequent to decreased personnel’s productivity due to absence from work [21,22].

Our Occupational Health Department, in collaboration with the Health Direction, plays a critical role in achieving higher immune coverage. According to international guidelines, the MMR vaccine is recommended and free for healthcare personnel without appropriate documentation about their vaccination status and without serological immunization, including students. [7,15]. Active immunization has a huge relevance in the fight against infectious diseases, which are an important concern for HCWs and especially for medical students.

## 5. Conclusions

Our results showed no change in vaccination coverage after the introduction of the last NPVP 2017–2019 and the following Law 119/2017. Only half of the students without protection against measles accepted to be vaccinated. The Occupational Health Department has the responsibility to sensitize at-risk personnel about the importance of vaccinations to increase immunity coverage by pre-employment screening and free vaccination campaigns. Establishing policies for measles vaccination for healthcare personnel is an important strategy to control virus circulation and should have high priority.

## Figures and Tables

**Table 1:** Characteristics of the samples in 2018 and 2017.

Characteristcs	2018	2017
Sample Size *N*	319	314
Age, Mean ± DS	25.7 ± 1.7	25.3 ± 1.5
Serologically Non-Immune *N* (%)	84 (26.3%)	85 (27.1%)
Serologically Non-Immune and Non-Vaccinated	68 (21.3%)	69 (22%)
Students Accepting Rescue Vaccination	35 (10.9%)	35 (11.1%)
